# Polydopamine-coupled NT_3_-derived oriented conductive scaffolds with immunomodulatory properties accelerate peripheral nerve regeneration

**DOI:** 10.4103/NRR.NRR-D-24-01544

**Published:** 2025-09-03

**Authors:** Xiaokun Chen, Jihai Xu, Ziyuan Yang, Jiahua Zhou, Feng Qin, Xueyuan Li, Miao Yu, Yanhua Wang, Ming Li, Xin Wang

**Affiliations:** 1 Department of Orthopedic Surgery, Ninth People’s Hospital Affiliated to Shanghai Jiao Tong University School of Medicine, Shanghai, China; 2 Department of Hand Surgery, Department of Plastic Reconstructive Surgery, Ningbo No.6 Hospital, Ningbo, Zhejiang Province, China; 3 Key Laboratory of Trauma and Neural Regeneration, Ministry of Education, Peking University, Beijing, China; 4 National Center for Trauma Medicine, Beijing, China; 5 Trauma Medicine Center, Peking University People’s Hospital, Beijing, China; 6 Department of Orthopedics and Trauma, Peking University People’s Hospital, Beijing, China; 7 Department of Hand Surgery, Ningbo No.6 Hospital, Ningbo, Zhejiang Province, China; 8 Department of Plastic Reconstructive Surgery, Ningbo No.6 Hospital, Ningbo, Zhejiang Province, China

**Keywords:** carbon nanotubes, electrospinning nerve catheter, immune regulation, neurotrophin-3, peripheral nerve regeneration

## Abstract

Peripheral nerve injury is a complex condition presenting significant clinical treatment challenges due to the limited regenerative capacity of peripheral nerves. Nerve conduits have been seen as a promising strategy to overcome the shortage of other treatment options (e.g., nerve graft). However, nerve regeneration occurs within a complex environment, and elaborate modulation is needed to meet repair requirements. The aim of this study was to investigate and explore a multifunctional nerve conduit with reactive oxygen species clearing, immune modulation to reshape the regenerative environment, and topographic cues and electrical signals to guide nerve growth. We developed an electroactive nerve guidance conduit composed of polylactic-glycolic acid and carbon nanotubes with an oriented structure using electrospinning and modified it with mussel-inspired polydopamine combining neurotrophin-3. The resulting nerve scaffold exhibited favorable orientation, electrical conductivity, and mechanical properties. Continuous release of neurotrophin-3 from the nerve conduit supported nerve regeneration throughout the repair process. *In vitro* assessments confirmed the cytocompatibility, reactive oxygen species scavenging, and immune regulation capabilities of the nerve scaffolds. In a rat sciatic nerve defect model, the nerve scaffolds effectively prevented muscle atrophy and promoted nerve regeneration and functional recovery over a 12-week period. These findings suggest that polydopamine-modified, electroactive, oriented nerve guidance conduits with multiple bioactive functions hold great promise for the repair of peripheral nerve injuries.

## Graphical abstract

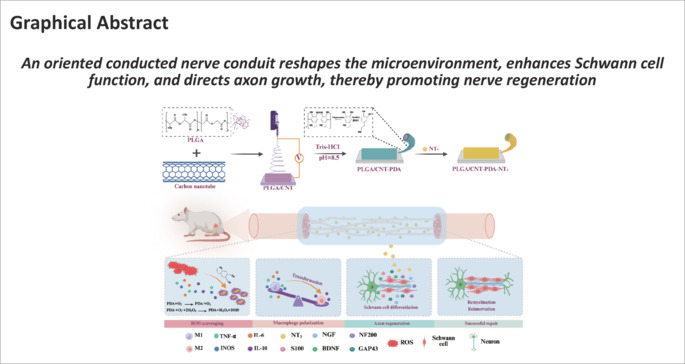

## Introduction

Peripheral nerve injury (PNI) is a common cause of persistent pain and long-term disability (Javeed et al., 2021), affecting approximately 1 million people worldwide each year. In addition to diminishing the quality of life of those affected, PNI also imposes significant economic burdens. Given its high prevalence, limited treatment options, and suboptimal clinical outcomes, PNI has remained a major challenge in clinical practice (Vijayavenkataraman, 2020; Singh et al., 2022). Nerve end-to-end anastomosis is commonly used to repair PNI involving small nerve gaps, while autografts are employed to address larger gaps. Although autografts are considered the “gold standard” clinical treatment for PNI, they are fraught with challenges, including a shortage of available autologous nerve grafts and complications at the donor site (Millesi, 2007). In recent years, tissue engineering has made significant strides, showing great promise in the treatment of PNI and the potential to replace autologous nerve transplants (Jiang et al., 2022).

Electrospun microfibers replicate the composition and structure of the extracellular matrix, providing topographical cues that guide peripheral nerve tissue growth. Uniaxially oriented PCL microfibers can direct Schwann cell (SC) migration and promote directional neurite extension from chicken embryo dorsal root ganglia (DRG), facilitating peripheral nerve regeneration (Murugan and Ramakrishna, 2007; Nune et al., 2019; Yang et al., 2022). Another key characteristic of nerve tissue and neurons is their electrophysiological activity, meaning that neuronal cell function can be regulated by applying low-intensity electrochemical currents in the millivolt range. Therefore, using conductive materials that can bridge the damaged ends of injured nerves and restore normal signal transmission is crucial for modulating nerve cell behavior and promoting the formation of functional neural connections. Carbon nanotubes (CNTs), classic electroactive biomaterials, have gained significant attention in the context of neural engineering applications due to their unique structure, electrical conductivity, and mechanical properties (Scapin et al., 2015; Lee et al., 2018). They are an effective conductive substrate, enabling transmission of vital electrical signals between neurons (Saberi et al., 2019).

Peripheral nerve regeneration is a complex process that occurs within a delicate microenvironment. Nerve growth factors and other bioactive substances play a crucial role in repairing injured nerves (Ding et al., 2024; Cong et al., 2025). In PNI, Schwann cells undergo dedifferentiation, allowing them to proliferate, generate new myelin, and secrete neurotrophin-3 (NT_3_) to promote axon regeneration (Jessen and Arthur-Farraj, 2019). NT_3_, a key member of the classical neurotrophic factor family, shares similarities with brain-derived neurotrophic factor (BDNF) and nerve growth factor (NGF) (Li et al., 2021). Research has shown that NT_3_ has a range of effects, including promoting neurite growth and myelination, as well as regulating synaptic plasticity, all of which are essential for the efficient repair of damaged nerves (Yalvac et al., 2016; Keefe et al., 2017). While electrospinning technology enables the integration of proteins into scaffolds, the organic solvents used in electrospinning precursor solutions can be toxic and negatively impact the biological activity of the proteins. As a result, NT_3_-loaded nerve guiding conduits may not achieve optimal biological effects.

Polydopamine (PDA) is a novel type of surface-modified biomimetic polymer that exhibits properties similar to those of proteins secreted by mussels (Huang et al., 2014). It offers several advantages, including strong adhesion, excellent biocompatibility, cell affinity, and biodegradability, and is widely used in various fields, including biomedicine (Liu et al., 2014; Cheng et al., 2019). Covalent attachment of polypeptides to PDA under mild reaction conditions, through mechanisms like Michael addition or the Schiff reaction, has been well documented (Bao et al., 2023; Guo et al., 2024). Therefore, the use of modified PDA, in combination with NT_3_, can overcome the challenges associated with NT_3_ loading and maintaining NT_3_ bioactivity during electrospinning. Notably, PDA’s rich array of chemical groups allows it to effectively remove reactive oxygen species (ROS), which play a critical role in preventing phenotypic transition of macrophages from the M1 to M2 state, a key factor in sustaining the inflammatory response (Qi et al., 2024). This property enables PDA to exert anti-inflammatory and immune-regulatory effects. However, to date, the synergistic effects of PDA bioactive coatings and electroactive, aligned microfibers on regulation of the regenerative immune microenvironment have not been thoroughly explored.

This study aimed to fabricate an electroactive, oriented neural scaffold using electrospinning technology. The scaffold’s surface was coated with PDA, which was covalently linked to NT_3_ through Schiff base or Michael addition reactions, resulting in a novel bioactive scaffold. The polylactic-glycolic acid (PLGA)/CNT-PDA-NT_3_ scaffold demonstrated excellent orientation, electrical conductivity, neurotrophic factor loading capacity, and sustained release properties. *In vitro* assessments were conducted to evaluate cell compatibility, the effects of the PLGA/CNT-PDA-NT_3_ scaffold on Schwann cell and PC12 cell growth, and the ability of the scaffold to scavenge ROS and regulate immune responses. *In vivo* experiments were conducted using a 10 mm rat sciatic nerve defect model to assess the scaffold’s effectiveness in promoting nerve regeneration and functional recovery, as well as measuring the expression of inflammatory factors and indicators of nerve regeneration. We believe this multifunctional nerve conduit holds promise for applications in promoting of in other electro-responsive tissues, such as the heart, muscle, and bone.

## Methods

### Preparation of polylactic-glycolic acid/carbon nanotube fibrous scaffolds

A 10% PLGA (Jinan Daigang Biomaterial Co., Ltd, Jinan, China) solution was prepared by dissolving 1 g of PLGA powder in 9 mL of hexafluoro-isopropanol. Subsequently, 30 mg of CNTs (XFNANO, Nanjing, China) was weighed and dispersed in 1 mL of hexafluoro-isopropanol (Sinopath, Beijing, China). The two solutions were thoroughly mixed and transferred into a 10-mL injection pump fitted with a 20-gauge needle. Electrospinning was performed under the following parameters: voltage +15 kV, flow rate 1.5 mL/h, using a rotating drum collector with a collection speed of 2000 r/min. The working distance was maintained at 15 cm. After electrospinning, the PLGA/CNT fiber scaffolds were collected and vacuum-dried overnight at room temperature.

### Fabrication of polydopamine-coated polylactic-glycolic acid/carbon nanotube scaffolds

The PLGA/CNT fibrous scaffold was placed in a 2 mg/mL dopamine solution (Sigma, St. Louis, MO, USA) prepared using 10 mM Tris-HCl buffer at a pH of 8.5. The PLGA/CNT scaffold was immersed in a 2 mg/mL dopamine solution (Sigma) prepared in 10 mM Tris-HCL buffer at pH 8.5 and incubated with continuous agitation at 100 r/min for 6 hours at room temperature. The resulting PLGA/CNT-PDA fiber scaffolds were washed three times with sterile deionized water and then freeze-dried.

### Characterization of the scaffolds

The surface morphology of the PLGA/CNT and PLGA/CNT-PDA fibers was analyzed by scanning electron microscopy (SEM) (JSM-7900F, JEOL, Tokyo, Japan). Energy-dispersive spectroscopy (EDS) and elemental mapping were used to investigate the chemical composition of the scaffolds. Fiber diameters were measured using Image-Pro Plus 6.0 software (Media Cybernetics, Rockville, MD, USA). Additionally, the water contact angles of the PLGA/CNT and PLGA/CNT-PDA scaffolds were determined using a contact angle measurement device (OCA20, DataPhysics, Filderstadt, Germany).

The tensile properties of the PLGA/CNT and PLGA/CNT-PDA scaffolds were evaluated by trimming the fibrous scaffolds into 3 × 5 cm^2^ samples, subjecting them to a load of 100 N, and stretching them at a rate of 10 mm/min three times using a mechanical testing machine (Solarbio, Beijing, China).

PLGA/CNT, PLGA/CNT-PDA, and PLGA/CNT-PDA-NT_3_ scaffold degradation was evaluated by weight loss analysis. After being incubated in phosphate-buffered saline (PBS) at 37°C for three months, the scaffolds were retrieved, vacuum-dried, and reweighed to determine the difference between their initial and final weights. The degradation rate was then calculated as the ratio of weight lost to the initial weight.

### Neurotrophin-3 loading and release assay

The sterilized PLGA/CNT and PLGA/CNT-PDA scaffolds were cut into 12 × 12 mm squares and immersed in 1 mL of a 1 μg/mL NT_3_ solution (Sinopath) for 12 hours at ambient temperature to obtain NT_3_-loaded scaffolds, referred to as PLGA/CNT-NT_3_ and PLGA/CNT-PDA-NT_3_. These NT_3_-loaded scaffolds were rinsed with deionized water and freeze-dried. The NT_3_ content within the scaffolds was measured using an enzyme-linked immunosorbent assay kit purchased from Solarbio. NT_3_ loading efficiency was determined by calculating the ratio of the NT_3_ incorporated into the scaffolds to the initial amount of NT_3_.

To mimic NT_3_, fluorescein isothiocyanate-labeled bovine serum albumin (Solarbio) was immobilized onto the fibrous scaffolds. Following fluorescein isothiocyanate-labeled bovine serum albumin loading, the scaffolds were thoroughly washed with deionized water. Fluorescein isothiocyanate-labeled bovine serum albumin distribution on the scaffolds was then analyzed by confocal laser scanning microscopy (CLSM) (TFS-SP8, Leica, Wetzlar, Germany).

To evaluate the scaffolds’ release profiles, 12 × 12 mm samples of the PLGA/CNT-NT_3_ and PLGA/CNT-PDA-NT_3_ scaffolds were immersed in 2 mL of PBS and incubated at 37°C with shaking at 100 r/min. At predetermined intervals (1, 3, 5, 7, 14, and 28 days), 1 mL of the supernatant was collected and replaced with an equal volume of PBS. The amount of NT_3_ released into the supernatant was quantified using an NT_3_ enzyme-linked immunosorbent assay kit (Solarbio). The cumulative release percentages were calculated and plotted over time to construct an NT_3_ release curve.

### Cell culture

PC12 cells (ATCC CRL-1721, Cell Bank of the Chinese Academy of Sciences, Shanghai, China) were cultured in a mixture containing 90% RPMI-1640 and 10% fetal bovine serum (FBS) (Thermo Fisher Scientific, Waltham, MA, USA). RSC96 Schwann cells (ATCC CRL-2765, Cell Bank of the Chinese Academy of Sciences) were seeded at a density of 300 cells/cm^2^ into standard culture plates or plates coated with the different fibrous scaffolds. These cells were maintained in high-glucose Dulbecco’s Modified Eagle’s Medium (DMEM; HyClone, Logan, UT, USA) supplemented with 10% FBS. All cell cultures were maintained at 37°C in a humidified atmosphere containing 5% CO_2_.

### RSC96 cell proliferation and morphology on scaffolds

RSC96 cell proliferation on the fibrous scaffolds was assessed by Cell Counting Kit-8 (CCK-8) assay. The cells were cultured on the scaffolds for 1, 3, and 5 days, after which the samples were rinsed with PBS and incubated with a 10% CCK-8 solution (Dojindo, Tokyo, Japan) for 2 hours. Then, the absorbance at 450 nm was measured using a microplate reader (SpectraMax M2, Molecular Devices, Sunnyvale, CA, USA) to quantify cell proliferation.

Following 3 days of culturing, Schwann cell morphology was analyzed by SEM and CLSM. For SEM, the samples were washed with PBS, followed by overnight immersion at 4°C in a 2.5% glutaraldehyde solution (Sigma). Subsequently, the samples were subjected to dehydration with a graded ethanol series, critical point drying, and gold sputter-coating (using a machine from Solarbio). For CLSM and quantitative analysis, the samples were first rinsed with PBS and fixed at 4°C overnight in 4% paraformaldehyde (Solarbio). Subsequently, the samples were permeabilized using 0.5% Triton X-100 (Solarbio), blocked with 5% BSA (Solarbio), and incubated overnight at 4°C with a rabbit anti-S100 antibody (1:200, HPA015768, Sigma). After washing, the samples were treated with Alexa Fluor 594-conjugated anti-rabbit IgG (1:200, R37119, ZSBG-Bio, Beijing, China) for 2 hours at room temperature. 4,6-Diamidino-2-phenylindole (DAPI; Sigma) was used for nuclear staining.

### Wound healing experiment

RSC96 cells were seeded into six-well plates at a density of 1 × 10^5^ cells per well. After 24 hours of culturing, a scratch wound was created using a 10-µL pipette tip. The cells were then cultured in conditioned medium (Solarbio) for another 48 hours, followed by two washes with PBS. Imaging was conducted by CLSM. Image-Pro Plus 6.0 software was employed to quantify the wound area.

### PC12 cell axon growth

Actin filaments were stained with phalloidin (Solarbio). Nuclei were stained with DAPI. The stained cells were then imaged by CLSM. Ten random images were captured for each sample, and the average and maximum axon lengths were determined using Image-Pro Plus 6.0. The fluorescence intensity of the stained axons was calculated, and their arrangement was analyzed.

### Reactive oxygen species scavenging assessment

The antioxidant properties of the materials were assessed *in vitro* using an ROS assay kit (BIO-ZL, Shanghai, China). RSC96 cells were inoculated at a concentration of 3 × 10^4^ cells/mL into 24-well plates containing the scaffolds and cultured for 72 hours. Then, the wells were rinsed with DMEM without phenol red. Next, 400 μL of 100 μM H_2_O_2_ was added to each well, and the plate was incubated at 37°C for 2 hours. Subsequently, the wells were washed twice to eliminate H_2_O_2_, a dichloro-dihydro-fluorescein diacetate probe (10 mM) from Solarbio was added, and the cells were cultured at 37°C in darkness for 20 minutes and washed twice. Finally, the cells were visualized by CLSM to measure the fluorescence levels.

The 1,1-diphenyl-2-picryl-hydrazyl (DPPH) test is a commonly employed method to evaluate the antioxidant properties of natural compounds. To test the scaffolds, 3 mL of DPPH solution (Aladdin, Shanghai, China) was combined with 2 mL of a methanol solution containing extracts of the PLGA/CNT scaffold, PLGA/CNT-PDA scaffold, or PLGA/CNT-PDA-NT_3_ scaffold, and the mixtures were incubated for 10 minutes at room temperature under low-light conditions. The absorbance of the solutions at 517 nm was measured using a Spectronic Genesys 5 spectrophotometer (Thermo Fisher Scientific).

CCK-8 assay was used to assess the ability of the scaffolds to protect RAW 264.7 macrophages from ROS-induced damage. RSC96, PC12, and RAW 264.7 (CL-0190, Procell Life Science & Technology Co., Ltd, Wuhan, China) cells were seeded into a 96-well plate at a density of 8 × 10^3^ cells per well. After 48 hours of incubation, the original growth medium was removed and replaced with fresh medium (DMEM supplemented with 10% fetal bovine serum (Solarbio)) containing 10 μm H_2_O_2_ and the various fibrous scaffolds. The plate was then incubated at 37°C for 5 hours without light, after which cell viability was evaluated by CCK-8 assay.

### Macrophage phenotype modulation

PLGA/CNT, PLGA/CNT-PDA, and PLGA/CNT-PDA-NT_3_ were placed in confocal petri dishes and sterilized with 75% ethanol and ultraviolet irradiation. Subsequently, 1 × 10^5^ RAW 264.7 macrophages were seeded into each plate, and the plates were incubated at 37°C overnight to facilitate cell adhesion. To induce M1 polarization, the cells were then exposed to lipopolysaccharide (100 ng/mL, Solarbio) and interferon-gamma (20 ng/mL, Solarbio) for 24 hours. Similarly, M2 polarization was induced with IL-4 (20 ng/mL, Solarbio) for 24 hours. Next, the cells were fixed with 4% paraformaldehyde, permeabilized with 0.5% Triton X-100 (Solarbio) and blocked with 1% BSA (Solarbio). Subsequently, they were subjected to nuclear/F-actin staining using TRITC-phalloidin/DAPI (Sigma) and visualized by CLSM.

### Quantitative reverse transcription-polymerase chain reaction

Total RNA was extracted from Schwann cells after 5 days of cultivation on the scaffolds using an RNA purification kit (ES Science, Shanghai, China). Subsequently, the RNA was reverse transcribed into cDNA using a reverse transcription kit (ABM, Vancouver, Canada). Quantitative real-time polymerase chain reaction (PCR) analysis was performed using a CFX96TM real-time PCR system (Bio-Rad, Hercules, CA, USA) and SYBR Green Realtime PCR Master Mix (TOYOBO, Osaka, Japan). Relative gene expression levels were determined using the 2^–ΔΔCt^ method and normalized to the housekeeping gene GADPH. **Additional Table 1** shows the primer sequences used in this study.

**Additional Table 1:** Primers used in this study for quantitative reverse transcription-polymerase chain reaction

Primer names	Forward sequences	Reverse primer sequences
*S100*	5'-ATGTCTGAGCTGGAGAAGG-3'	5'-TCACTCATGTTCAAAGAACTC-3'
*NGF*	5'-ACAGGCAGAACCGTACACAG-3'	5'-CTATTGGTTCAGCAGGGGCA-3'
*BDNF*	5'-TCCGAGAGCTTTGTGTGGAC-3'	5'-TTTGCTTCTTTCATGGGCGC-3'
*TNF-α (Tnf)*	5'-GGCAGGTTCTGTCCCTTTCA-3ʹ	5'-TCTTCTGCCAGTTCCACGTC-3ʹ
*iNOS (Nos2)*	5'-GCCAACATGCTACTGGAGGT-3ʹ	5'-TCCAGGATGTTGTAGCGCTG-3ʹ
*IL-6*	5'-TTCTTGGGACTGATGCTGGTG-3ʹ	5'-CACAACTCTTTTCTCATTTCCACGA-3ʹ
*IL-10*	5'-CCAAGCCTTATCGGAAATGA-3 ʹ	5'-TCCTGAGGGTCTTCAGCTTC-3ʹ
*NF200 (Nefh)*	5ʹ -ACAT GGCCTCCT ACCAGGAT-3ʹ	5ʹ-TCTTGACGTTGAGCAGGTCC-3ʹ
*GAP43*	5ʹ-GGAGAAGGATGATGCTCCCG-3ʹ	5ʹ-TCGCCCTTCTTCTCCTCAGA-3ʹ
*GAPDH*	5'-GAAGGGCATCTTGGGCTACAC-3'	5'-GTTGTCATTGAGAGCAATGCCA-3'

BDNF: Brain-derived neurotrophic factor; GAP43: growth associated protein 43; GAPDH: glyceraldehyde 3-phosphate dehydrogenase; IL: interleukin; iNOS inducible nitric oxide synthase; NF200: neurofilament-200; NGF: nerve growth factor; TNF-α: tumor necrosis factor-α.

### *In vivo* biocompatibility assessment

The animal procedures were approved by the Animal Ethics Committee of Peking University People’s Hospital on October 25, 2023 (approval No. 2023PHE062) and conducted in strict accordance with the National Institutes of Health Guide for the Care and Use of Laboratory Animals (8^th^ ed., National Research Council, 2011). Female SD rats (age: 6–8 weeks), each weighing between 200 and 220 g and free of specific pathogens, were sourced from Beijing SPF Biotechnology Co., Ltd (license No. SCXK (Jing) 2024-0001). Only female rats were used, as they are less aggressive than male rats, and are therefore less likely to incur additional injuries that may impact nerve repair. In addition, female rats were used in a previous study (Pi et al., 2022). Moreover, there is little evidence suggesting that sex differences make female animals unsuitable as models (Zucker and Beery, 2010). All SD rats were housed in a sterile environment, with 12 hours of light and 12 hours of darkness, at a constant temperature of 20°C. They had unrestricted access to both standard laboratory feed and water throughout the study period. The rats were randomly assigned to one of four groups (*n* = 6/group): PLGA/CNT, PLGA/CNT-PDA, PLGA/CNT-PDA-NT_3_, and autograft. To assess histocompatibility, the rats were anesthetized by isoflurane inhalation (3% for induction, 1.5% for maintenance, RWD Life Science Co., Ltd, Shenzhen, China), and 10 × 5 mm^2^ samples of each fibrous scaffold were implanted into the back muscles. The samples were retrieved from the muscle tissue on days 3, 7, and 14. The harvested samples were fixed for 24 hours in 4% paraformaldehyde, dehydrated for 48 hours in a 30% sucrose solution, stored at –80°C, and sliced for analysis. Hematoxylin and eosin (HE) staining was performed, and the inflammatory response was assessed via immunofluorescence staining for interleukin-6 (IL-6) and tumor necrosis factor-α (TNF-α).

### Surgical procedures

The PCL/CNT, PCL/CNT-PDA, and PLGA/CNT-PDA-NT_3_ fibrous scaffolds were fashioned into nerve conduits measuring 12 mm in length with a 2-mm inner diameter. Anesthesia was induced in all rats through inhalation of 3% isoflurane. Following shaving and disinfection, the right sciatic nerve was exposed, and a 10-mm nerve segment was excised at the mid-femur level. In the autograft group, the 10-mm nerve segment was preserved, re-bridged, and sutured back to the nerve stumps using 8/0 nylon sutures. In the other groups, the proximal and distal nerve remnants were sutured 1 mm deep into the nerve conduits with 8/0 nylon sutures. Muscle and skin incisions in all groups were subsequently closed using 4/0 nylon sutures.

### Walking track examination

The function of the regenerated sciatic nerves was evaluated 1, 2, and 3 months after the surgical procedure by walking track analysis. The rat gaits were captured using a CatWalk Gait Analysis System (Noldus, Wageningen, The Netherlands). Several parameters were evaluated, including experimental toe spread (ETS), normal toe spread (NTS), experimental paw length (EPL), normal paw length (NPL), experimental intermediary toe spread (EIT), and normal intermediary toe spread (NIT). The sciatic functional index (SFI) was determined using the following formula (Pi et al., 2022):


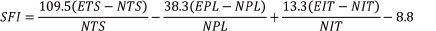


### Specimen collection and records

Three months post-surgery, all rats were euthanized with 40% CO_2_. Photographs were taken of the gastrocnemius muscle, and its weight was recorded. The control group was denoted as *Mc*, while the experimental group was labeled as *Ms*. The wet-weight ratio of the gastrocnemius muscle was calculated as follows (Xiong et al., 2023):


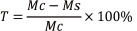


The middle segment of the sciatic nerve in the right gastrocnemius muscle and the gastrocnemius muscle itself were harvested and preserved at 4°C in 4% paraformaldehyde. In addition, distal segments of the nerve were collected and fixed at 4°C in 2.5% glutaraldehyde.

### Electrophysiological examination

Electrophysiological tests were performed on the rats using a Biological Function Assay System (BL-420S, Keypoint, Nørresundby, Denmark) 3 months post-implantation. The stimulation electrode was positioned approximately 2 mm proximal to the graft site, and the receiving electrode was placed at a distal site to record nerve conduction velocity and complex muscle action potential.

### Histological assessment of muscle

The harvested muscles were embedded in paraffin wax and sliced into transverse sections. Ten sections from each group were randomly selected for HE staining (Solarbio). Imaging was performed using a Leica DM4B microscope. For each section, six random fields of view were selected, and the cross-sectional area of the muscle fibers was measured using Image-Pro Plus 6.0.

### Immunofluorescence analysis of regenerated nerves

The midsection of the sciatic nerve was embedded in optimal cutting temperature compound 2 weeks post-implantation and sliced into 12-μm-thick sections. Double immunofluorescence staining was performed (*n* = 10/group). Mouse anti-neurofilament-200 (NF200; 1:200, N0142) and rabbit anti-S100 (1:200, HPA015768), both purchased from Sigma, were used as the primary antibodies. Alexa Fluor 488 anti-mouse IgG (1:200, A11006) and Alexa Fluor 594 anti-rabbit IgG (1:200, R37119), purchased from ZSBG-Bio, were used as the secondary antibodies. DAPI was used for nuclear staining, and images were acquired by CLSM. Ten random fields from each slide were visualized, and the NF200- and S100-positive area was calculated using Image-Pro Plus 6.0.

TNF-α and IL-6 immunofluorescence was used to assess inflammation in the regenerated nerve tissue. M1 and M2 macrophages were detected using anti-CD86 antibodies and anti-CD206 antibodies, respectively. The cells were imaged by CLSM (TCS-SP8, Leica), and fluorescence intensity was analyzed using Image-Pro Plus 6.0.

### Toluidine blue staining and transmission electron microscopy

The distal segment of the regenerated nerve was embedded in resin and then sliced into 700-nm semi-thin sections, as well as 70-nm ultra-thin sections. Toluidine blue (Solarbio) staining was performed (*n* = 10/group), and the sections were imaged via transmission electron microscopy. Ten random visual fields were analyzed for each section. Myelinated nerve density was measured using Image-Pro Plus 6.0. Similarly, sections were stained with uranyl acetate-lead citrate, images were obtained by transmission electron microscopy (JEM-1400, Leica), and the images were analyzed using ImageJ software (Media Cybernetics) to measure myelin thickness and the diameters of the myelinated nerve fibers.

### Statistical analysis

Data are expressed as mean ± SD. Differences between two groups were analyzed by Student’s *t*-test, while multiple groups were compared by one-way analysis of variance followed by Tukey’s *post hoc* test. All statistical analyses were carried out using SPSS 22.0 (IBM Corp., Armonk, NY, USA). *P*-values less than 0.05 were considered statistically significant.

## Results

### Preparation and characterization of the scaffolds

**Figure 1:**
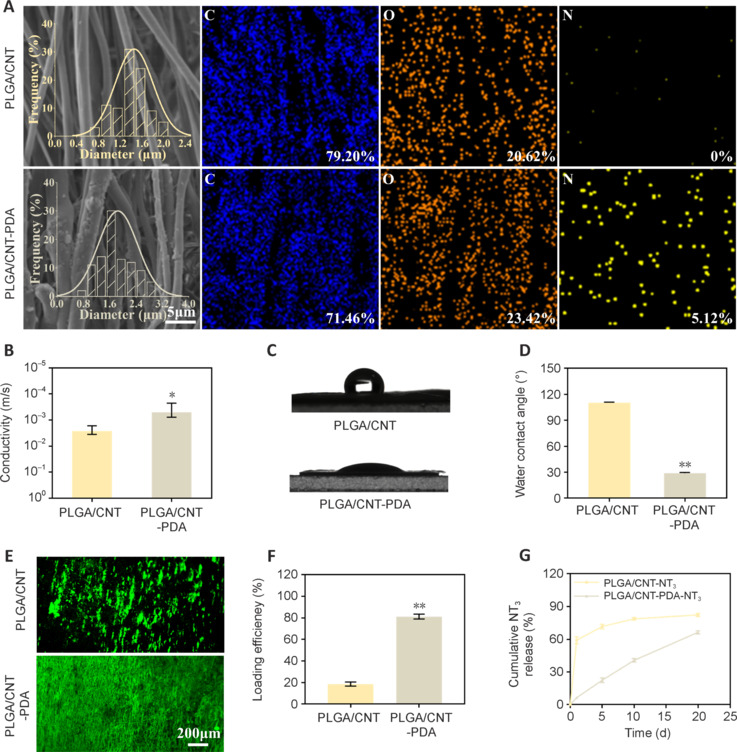
Preparation and characterization of PLGA/CNT-PDA-NT_3_. (A) SEM images of PLGA/CNT and PLGA/CNT-PDA fibers (including fiber diameter distribution, scale bar: 5 μm) and energy dispersion spectroscopy (EDS) analysis of carbon, nitrogen, and oxygen. (B) PLGA/CNT, PLGA/CNT-PDA, and PLGA/CNT-PDA-NT_3_ conductivity. (C, D) PLGA/CNT and PLGA/CNT-PDA water contact angles. (E) CLSM images of the PLGA/CNT and PLGA/CNT-PDA scaffolds loaded with luorescein isothiocyanate-labeled bovine serum albumin (scale bar: 200 μm). (F) NT_3_ loading capacity of PLGA/CNT and PLGA/CNT-PDA. (G) PLGA/CNT and PLGA/CNT-PDA scaffold NT_3_ release curves. Data are expressed as mean ± SD (*n* = 3). *P < 0.05, **P < 0.01, *vs*. PLGA/CNT (Student’s *t*-test). CNT: Carbon nanotube; NT_3_: neurotrophin-3; PDA: polydopamine; PLGA: polylactic-glycolic acid.

The structures of the successfully prepared fibrous scaffolds, as visualized by SEM, are shown in **Figure 1A**. It can be clearly seen that the surface of the scaffolds loaded with PDA had a rougher texture than those without PDA loading. Additionally, the average diameter of the fibrous scaffold increased from 1.458 μm to 1.848 μm when loaded with PDA (diameter distribution diagram showed in **Figure 1A**). The distributions of carbon, nitrogen, and oxygen in the PLGA/CNT and PLGA/CNT-PDA scaffolds were obtained via elemental analysis and EDS energy dispersion spectra. The results indicated a significantly higher content of nitrogen in PLGA/CNT-PDA compared with PLGA/CNT (**Figure 1A**), suggesting successful PDA decoration. The PLGA/CNT and PLGA/CNT-PDA scaffolds both exhibited good electrical conductivity (**Figure 1B**). The electrical conductivity of the PDA-coated scaffold was maintained 10−3 S/cm, which is sufficient to stimulate nerve cell proliferation and differentiation because of the low *in vivo* microcurrent intensity (Guimard et al., 2007; Zhang et al., 2010). The elastic modulus was determined through repeated tensile property tests (**Additional Figure 1**) and revealed no significant differences in tensile properties among the various fibrous scaffolds, indicating that PLGA/CNT maintained good mechanical properties after PDA and NT_3_ loading. Studies have shown that PLGA is a suitable material for nerve conduits (Zhao et al., 2016). PLGA has an optimal degradation time, allowing it to provide long-term mechanical support for nerve regeneration without hindering nerve repair (Pozzobon et al., 2021). *In vitro* degradation rate analysis (**Additional Figure 2**) showed no significant differences among the different fibrous scaffolds, suggesting that they maintained a low degradation rate after PDA and NT_3_ loading. The degradation rate was slow, and the time span covered the repair period, ensuring that the structure and conductivity were maintained throughout the repair phase, thereby supporting prolonged drug release and nerve regeneration.

Previous research has confirmed the significance of hydrophilicity of scaffolds in peripheral nerve tissue regeneration and repair (Guo et al., 2016). This property plays a crucial role in maintaining a moist environment, thereby facilitating Schwann cell and neuron proliferation and migration. To assess the hydrophilicity of PDA-coated fibrous scaffolds, their water contact angles were determined. The water contact angles for PLGA/CNT and PLGA/CNT-PDA were 110.3° and 58.89°, respectively (**Figure 1C** and **D**). The significant variance (*P* < 0.01) between the two values suggests that PDA modification substantially enhanced hydrophilicity. This improvement is advantageous for nerve tissue and cell regeneration.

CLSM imaging revealed significantly higher surface fluorescence intensity of the PLGA/CNT-PDA fibers compared with the PLGA/CNT scaffold (**Figure 1E**), suggesting that the PDA-modified fibrous scaffold could bind more protein factors and had enhanced loading efficiency compared with the PLGA/CNT scaffold. Next, NT_3_ loading and release were quantified by enzyme-linked immunosorbent assay. As depicted in **Figure 1F**, the loading capacity of PLGA/CNT-PDA was notably higher than that of PLGA/CNT (81.1 and 18.6, respectively, *P* < 0.01), which is consistent with the immunofluorescence staining results shown in **Figure 1E**. Additionally, NT_3_ was released more slowly and steadily from the PLGA/CNT-PDA scaffold than from the PLGA/CNT scaffold (**Figure 1G**); this sustained release aligns well with the prolonged amount of time required for nerve injury regeneration and repair.

### Schwann cell adhesion, proliferation, and migration on scaffolds

**Figure 2:**
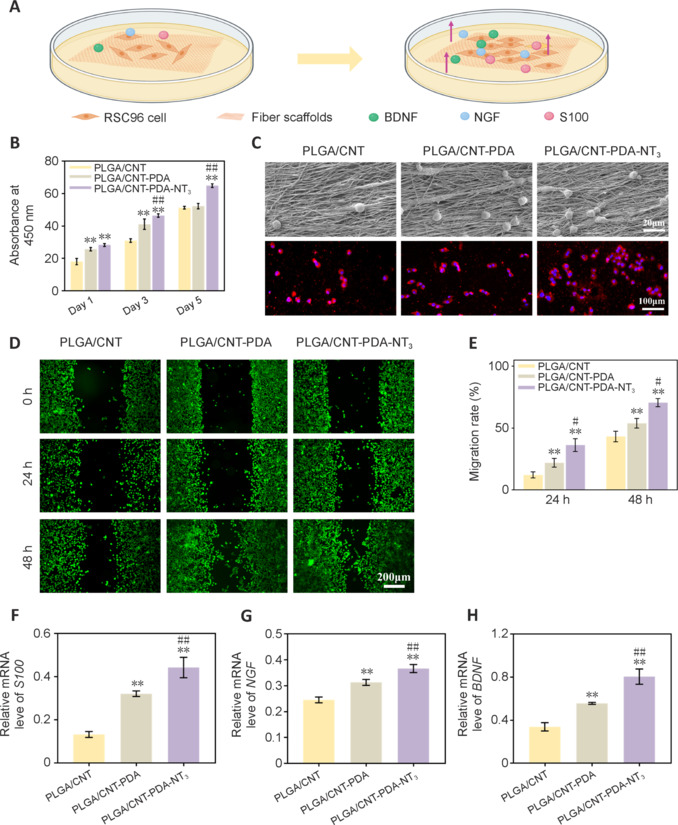
Effect of PLGA/CNT-PDA-NT_3_ on Schwann cell proliferation and migration. (A) Schematic diagram showing the mechanism by which PLGA/CNT-PDA-NT_3_ promotes Schwann cell growth. (B) RSC96 cell proliferation on PLGA/CNT, PLGA/CNT-PDA, and PLGA/CNT-PDA-NT_3_ scaffolds (*n* = 3). (C) Scanning electron microscopy and CLSM images of RSC96 cells. Red staining indicates S100 in the CLSM images. Nuclei were stained blue with 4,6-diamidino-2-phenylindole (scale bars: 10 μm, 100 μm). (D) RSC96 cell scratch test on different scaffolds (scale bar: 200 μm). (E) Wound healing rates from the cell scratch assay (*n* = 6). (F–H) Quantitative polymerase chain reaction analysis of *S100*, *NGF*, and *BDNF* expression. Data are expressed as mean ± SD (*n* = 3 in B and 6 in E). **P < 0.01, *vs.* PLGA/CNT; #*P* < 0.05, ##*P* < 0.01, *vs.* PLGA/CNT-PDA (one-way analysis of variance followed by Tukey’s *post hoc* test). BDNF: Brain-derived neurotrophic factor; CLSM: confocal laser scanning microscopy; CNT: carbon nanotube; NGF: nerve growth factor; NT_3_: neurotrophin-3; PDA: polydopamine; PLGA: polylactic-glycolic acid.

Schwann cells, a type of glial cell, play a crucial role in PNI regeneration and repair, as they cover a significant portion of axonal surfaces (Qu et al., 2021). Following initial injury, Schwann cells undergo trans-differentiation, transitioning from myelin-producing cells to repair cells that support nerve regeneration (Scheib and Höke, 2013). As shown in **Figure 2A**, we assessed the proliferation, migration, and protein expression of RSC96 cells *in vitro*. Schwann cell viability was determined by CCK-8 assay. As shown in **Figure 2B**, we observed a gradual increase in RSC96 cell numbers on days 1, 3, and 5. Notably, RSC96 cell viability on the PLGA/CNT scaffold was significantly lower than that on the PLGA/CNT-PDA and PLGA/CNT-PDA-NT_3_ scaffolds on days 1 and 3. Furthermore, the viability of RSC96 cells cultured on the PLGA/CNT-PDA-NT_3_ scaffold was notably higher on days 3 and 5 than that of cells cultured on the other scaffolds (*P* < 0.01). These findings suggest that the PLGA/CNT-PDA-NT_3_ scaffold effectively enhances Schwann cell growth. The SEM images in **Figure 2C** illustrate RSC96 cell morphology on the fibrous scaffolds, showing that the cells aligned along the fibers and acquire a spindle-shaped appearance. CLSM imaging revealed that a majority of the RSC96 cells adhered to and proliferated on the PLGA/CNT-PDA-NT_3_ scaffold, indicating that the oriented structure of the electrospun scaffold, along with the hydrophilic properties and NT_3_ release facilitated by the PDA coating, promoted Schwann cell adhesion and growth on the fabricated scaffolds.

In a cell scratch test, the Schwann cells exhibited the greatest degree of migration and proliferation on the PLGA/CNT-PDA-NT_3_ scaffold compared with the other scaffolds. The wound healing rate of Schwann cells grown on the PLGA/CNT-PDA-NT_3_ scaffold significantly exceeded that of cells grown on the other two scaffolds at 24 and 48 hours (**Figure 2D** and **E**). Real-time quantitative PCR analysis confirmed that the PLGA/CNT-PDA-NT_3_ fibrous scaffold upregulated *S100*, *NGF*, and *BDNF* expression in Schwann cells (**Figure 2F–H**) compared with the other two scaffolds (*P* < 0.05). Notably, S100 is a specific marker of Schwann cells, suggesting that the PLGA/CNT-PDA-NT_3_ scaffold enhanced Schwann cell growth, proliferation, and myelination of Schwann cells by inducing NGF and BDNF expression. The role of NGF has been extensively investigated due to its widespread presence in healthy neurons. In injured nerves, NGF expression increases significantly, which plays a crucial role in promoting neuron growth and survival (Alvites et al., 2018). BDNF regulates synaptic function and may enhance nerve preservation and regeneration (Wang et al., 2019).

Schwann cells cultured on the PLGA/CNT-PDA-NT_3_ scaffold exhibited a spindle-shaped, orderly arrangement. Previous research has demonstrated that an increase in scaffold surface roughness enhances cell adhesion rates (Deligianni et al., 2001). As depicted in **Figure 1A**, PDA modification enhanced the surface roughness of the fibrous scaffolds. PDA is known as “biological glue” because it contains catechol and abundant chemical groups that promote cell adhesion and growth. The distinctive electrical properties of CNTs, in conjunction with the multifaceted biological functions of PDA, synergistically promote Schwann cell adhesion, proliferation, and differentiation, as well as increasing their secretion of various growth factors.

### PC12 cell axon growth and differentiation on scaffolds

**Figure 3:**
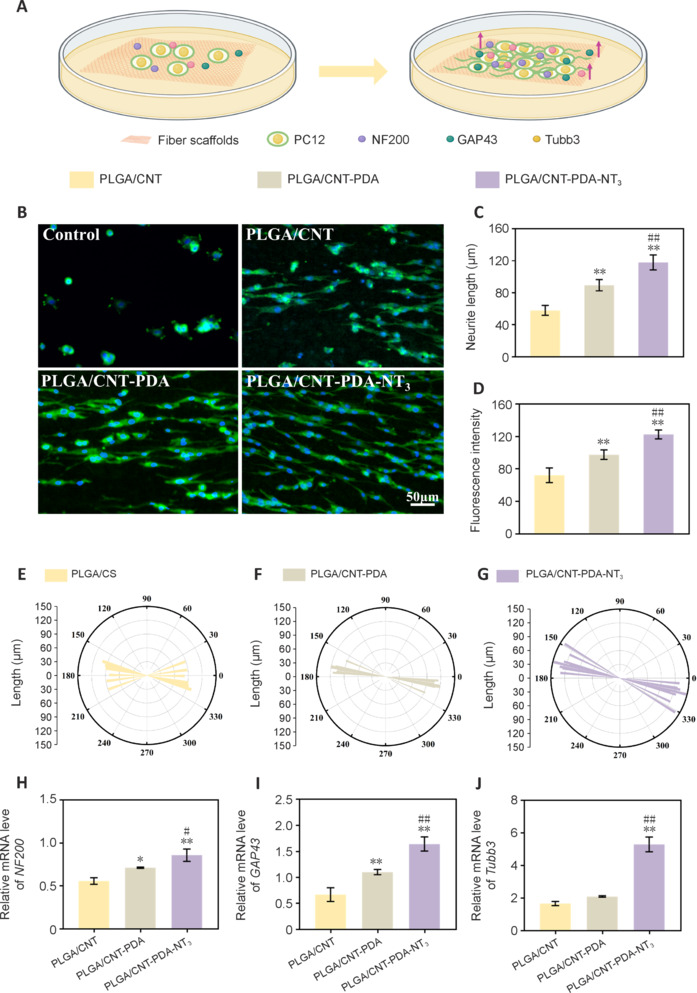
Effect of PLGA/CNT-PDA-NT_3_ on PC12 cell growth. (A) Schematic diagram of the mechanism through which neural conduits promote PC12 cell axon growth. (B) CLSM images of PC12 cells. DAPI staining is shown in blue, and phalloidin staining is shown in green (scale bar: 200 μm). (C) Average PC12 cell axon length (*n* = 6). (D) Quantitative analysis of immunofluorescence intensity (*n* = 6). (E–G) Polarity histogram of the directional distribution of PC12 cell axons on scaffolds. (H–J) Relative gene expression levels of neuronal markers (*NF200*, *GAP43*, and *Tubb3*), as determined by quantitative reverse transcription-polymerase chain reaction. Data are expressed as mean ± SD (*n* = 3). *P < 0.05, **P < 0.01, *vs*. PLGA/CNT; #*P* < 0.05, ##*P* < 0.01, *vs*. PLGA/CNT-PDA (one-way analysis of variance followed by Tukey’s *post hoc* test). CLSM: Confocal laser scanning microscopy; CNT: carbon nanotube; GAP43: growth associated protein 43; NF200: neurofilament-200; NT_3_: neurotrophin-3; PDA: polydopamine; PLGA: polylactic-glycolic acid.

PC12 cells are widely used in neurobiology research, as they possess features of mature dopaminergic neurons. The effects of our nerve conduits on PC12 cells were investigated, as shown in **Figure 3A**. of the PC12 cell cytoskeletal staining results are depicted in **Figure 3B**. In the control group, PC12 cells exhibited a random and irregular arrangement, whereas cells grown on the PLGA/CNT and PLGA/CNT-PDA scaffolds displayed a more regular pattern. Notably, PC12 cells cultured on PLGA/CNT-PDA-NT_3_ exhibited a highly organized and oriented structure, with the highest cell density. Next, we assessed the average axon length (**Figure 3C**), fluorescence intensity (**Figure 3D**), and arrangement (**Figure 3E–G**) of PC12 cells grown on the different scaffolds. Neurite length was measured to assess the level of PC12 cell differentiation. The average axon length was 58.09 μm in the PLGA/CNT group, while for PLGA/CNT-PDA, PLGA/CNT-PDA-NT_3_ scaffolds, it significantly increased to 89.52 μm and 117.9 μm, respectively (*P* < 0.01). The longest axon length was observed in the PLGA/CNT-PDA-NT_3_ group (*P* < 0.01). The fluorescence intensity from PLGA/CNT to PLGA/CNT-PDA to PLGA/CNT-PDA-NT_3_, with the latter exhibiting the highest intensity (*P* < 0.01). These results suggest that PC12 cells displayed favorable adhesion, growth, and differentiation on PLGA/CNT-PDA-NT_3_ scaffolds. Moreover, all three groups demonstrated good cell alignment, with the PLGA/CNT-PDA group showing the best alignment.

To further investigate the mechanism by which PLGA/CNT-PDA-NT_3_ facilitates nerve cell growth, *NF200*, growth associated protein 43 (*GAP43*), and *Tubb3* expression levels were assessed by real-time quantitative PCR. NF200 is a cytoskeletal protein found in nerve cells, while GAP43 is a neuron-specific protein located on the axonal membrane that plays a role in neuronal growth, synapse formation, and nerve cell regeneration. The relative expression levels of *NF200* by PC12 cells grown on PLGA/CNT, PLGA/CNT-PDA, and PLGA/CNT-PDA-NT_3_ were 0.5593, 0.6157, and 0.8600, respectively (**Figure 3H**). Notably, *NF200* expression was highest in the PLGA/CNT-PDA-NT_3_ group (*P* < 0.01). Similarly, *GAP43* and *Tubb3* expression was significantly higher in PC12 cells grown on the PLGA/CNT-PDA-NT_3_ scaffold compared with the other two scaffolds (*P* < 0.01; **Figure 3I** and **J**). These findings suggest that PLGA/CNT-PDA-NT_3_ effectively upregulates *NF200*, *GAP43*, *Tubb3* expression in PC12 cells, thereby promoting neuronal maturation and axon regeneration.

### The effects of inflammation inhibition and modulation *in vitro*

**Figure 4:**
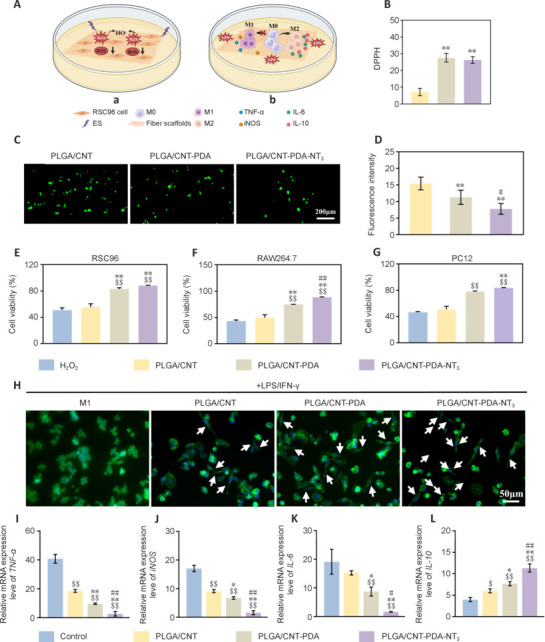
Antioxidant and immunomodulatory effects of PLGA/CNT-PDA-NT_3_. (A) Schematic diagram of ROS scavenging and macrophage phenotype induction by the fibrous scaffolds. (B) DPPH test of antioxidant capacity (*n* = 3). (C) ROS probe test (scale bar: 200 μm). (D) Quantitative analysis of ROS fluorescence intensity (*n* = 3). (E–G) Survival rates of RSC96, PC12, and RAW264.7 cells exposed to 100 μM H_2_O_2_, as measured by Cell Counting Kit-8 (*n* = 3). (H) Fluorescence images showing the cytoskeletons and nuclei of RAW 264.7 macrophages cultured for 24 hours under normal conditions or on fibrous scaffolds. Arrows indicate cells with M2 morphology (scale bar: 50 μm). (I–L) *TNF-α*, *iNOS*, *IL-6*, *IL-10*, and mRNA expression in RAW264.7 cells, as determined by quantitative reverse transcription-polymerase chain reaction. Data are expressed as mean ± SD (*n* = 3). *P < 0.05, **P < 0.01, *vs*. PLGA/CNT; #*P* < 0.05, ##*P* < 0.01, *vs*. PLGA/CNT-PDA; $$*P* < 0.01, *vs*. control /H_2_O_2_ group (one-way analysis of variance followed by Tukey’s *post hoc* test). CNT: Carbon nanotube; IL: interleukin; iNOS: inducible nitric oxide synthase; NT_3_: neurotrophin-3; PDA: polydopamine; PLGA: polylactic-glycolic acid; ROS: reactive oxygen species; TNF-α: tumor necrosis factor-α.

During tissue repair, macrophages induce inflammation by releasing ROS and defending against bacterial intrusion (Huber-Lang et al., 2018). However, sustained inflammation can cause oxidative stress, resulting in cellular damage that hinders tissue repair and nerve regeneration (Yao et al., 2019). Numerous studies have demonstrated that PDA, which is characterized by abundant functional groups and consequent reducibility (e.g., catechol and imine groups), could help clear various ROS in various circumstances (Jodko-Piórecka et al., 2015; Zhou et al., 2019). As shown in **Figure 4A**, we next explored the ability of our scaffolds to promote ROS scavenging and induce a macrophage phenotype shift. Antioxidant properties are commonly assessed using the DPPH test. We found that PLGA/CNT-PDA and PLGA/CNT-PDA-NT_3_ exhibited remarkably greater antioxidant capabilities than PLGA/CNT (*P* < 0.01) (**Figure 4B**), which we attributed to the abundant functional groups of PDA. The ROS probe test results are presented in **Figure 4C** and **D**. The ROS levels of PLGA/CNT-PDA and PLGA/CNT-PDA-NT_3_ were lower than those of PLGA/CNT (*P* < 0.01), indicating effective reduction of ROS by the PDA coating. Additionally, **Figure 4E–G** illustrates the survival rates of RSC96, PC12, and RAW264.7 cells on the various fibrous scaffolds when exposed to 10 μm H_2_O_2_. The cells exhibited higher survival rates on PLGA/CNT-PDA and PLGA/CNT-PDA-NT_3_ compared with PLGA/CNT (*P* < 0.01), demonstrating the superior antioxidant activity of PLGA/CNT-PDA-NT_3_ and its protective effects on Schwann cells, neurons, and macrophages.

Schwann cells and neurons play crucial roles in nerve regeneration, and macrophage migration and influx are pivotal steps in the PNI repair process (Barrette et al., 2008). Local cells surrounding the injury site are responsible for clearing axon residue and myelin fragments, as well as recruiting macrophages (Reichert et al., 1994), thereby creating a conducive environment for axon regeneration. In addition to phagocytosis and removal of residues, macrophages also produce growth factors and help regulate the extracellular matrix (Mueller et al., 2003). Macrophages are categorized into two distinct phenotypes: M1 and M2. Previous research has confirmed the impact of the two different macrophage phenotypes on nerve regeneration. M1 macrophages mainly exhibit pro-inflammatory effects, secreting TNF-α, IL-6, and other inflammatory factors, while M2 macrophages primarily exert anti-inflammatory functions (Mosser and Edwards, 2008). The inflammatory response in the early stages of tissue damage repair eliminates damaged cells and tissues; however, an excessively robust inflammatory reaction can cause cellular damage and hinder the repair process. Consequently, we assessed macrophage phenotypes *in vitro*. RAW264.7 cells cultured on various fibrous scaffolds exhibited diverse reactions to lipopolysaccharide exposure. As illustrated in **Figure 4H**, RAW264.7 cultured on PLGA/CNT-PDA and PLGA/CNT-PDA-NT_3_ scaffolds displayed a spindle-shaped morphology resembling that of M2 macrophages, contrasting with the spread-out, pseudopodium-rich structure of M1 macrophages, which suggested a shift towards M2 macrophage activation. Some of the RAW264.7 cells grown on the PLGA/CNT scaffold also exhibited this M2-like morphology. PCR analysis (**Figure 4I–L**) indicated that IL-6, inducible nitric oxide synthase (iNOS), and TNF-α expression levels in macrophages grown on the PLGA/CNT-PDA and PLGA/CNT-PDA-NT_3_ scaffolds were notably lower than those in macrophages grown on the PLGA/CNT scaffold (*P* < 0.01).

### Biocompatibility of polylactic-glycolic acid/carbon nanotube-polydopamine-neurotrophin-3 scaffolds *in vivo*

**Figure 5:**
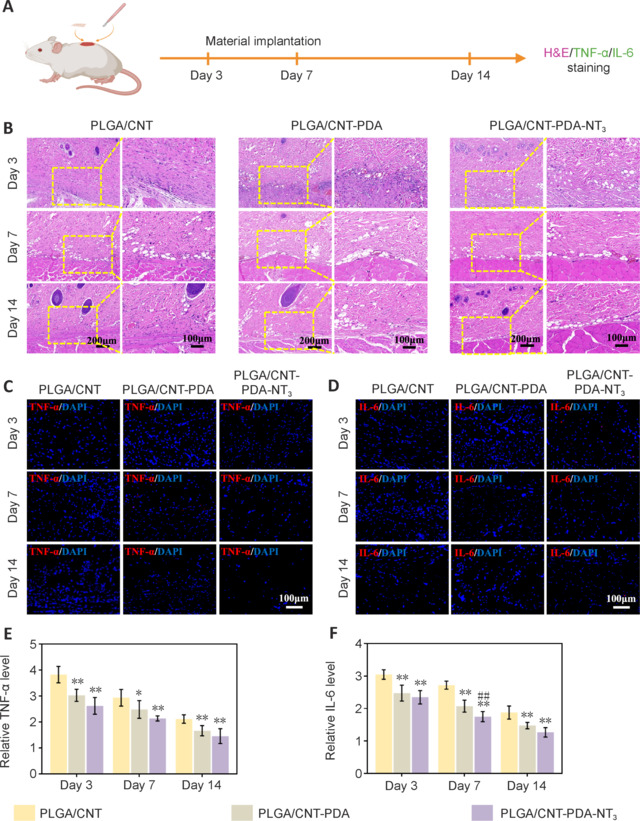
Immunohistochemical analysis of subcutaneous wounds embedded with PLGA/CNT, PLGA/CNT-PDA, or PLGA/CNT-PDA-NT_3_ in rats on days 3, 7, and 14. (A) Animal experiment timeline. (B) Hematoxylin and eosin staining of the wound tissue on days 3, 7, and 14 after embedding (scale bars: 200 μm, 100 μm). (C, D) TNF-α/DAPI and IL-6/DAPI staining of the wound tissue 3, 7, and 14 days after embedding (scale bar: 100 μm). (E) TNF-α fluorescence intensity. (F) IL-6 fluorescence intensity. Data are expressed as mean ± SD (*n* = 3). *P < 0.05, **P < 0.01, *vs*. PLGA/CNT; ##*P* < 0.01, *vs*. PLGA/CNT-PDA (one-way analysis of variance followed by Tukey’s *post hoc* test). CNT: Carbon nanotube; DAPI: 4,6-diamidino-2-phenylindole; IL: interleukin; NT_3_: neurotrophin-3; PDA: polydopamine; PLGA: polylactic-glycolic acid; TNF-α: tumor necrosis factor-α.

A schematic of the animal experiments is shown in **Figure 5A**. HE staining of wound tissue after subcutaneous embedding of the materials in animals (**Figure 5B**) revealed that the PLGA/CNT-PDA and PLGA/CNT-PDA-NT_3_ groups exhibited reduced inflammation and enhanced healing effects compared with the PLGA/CNT group. Immunofluorescence staining and analysis of TNF-α (**Figure 5C** and **E**) and IL-6 (**Figure 5D** and **F**) expression levels indicated a decrease in the expression of inflammatory factors across all groups over time, with a noticeable declining trend in all groups on days 3, 7, and 14. In particular, the PLGA/CNT-PDA-NT_3_ group mounted the mildest inflammatory response.

### Functional assessment of the sciatic nerve and histological analysis of the gastrocnemius muscle

**Figure 6:**
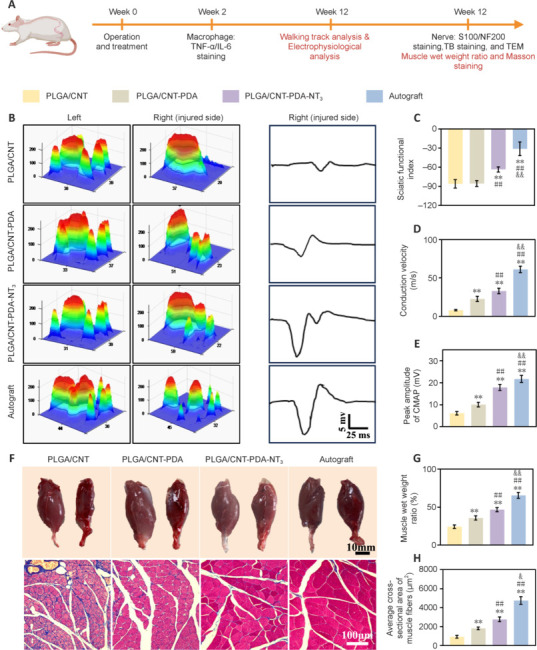
Functional and histological analysis of the sciatic nerve. (A) Schematic diagram of the effect of PLGA/CNT-PDA-NT_3_ on wound healing in a rat sciatic nerve defect model. (B) 3D distribution of plantar pressure. (C) Sciatic functional index evaluation. (D) Conduction velocity. (E) Peak CMAP amplitude. (F) Masson staining of gastrocnemius muscle cross-sections (scale bars: 10 μm, 100 μm). (G) Wet weight of the gastrocnemius muscle. (H) Cross-sectional area of gastrocnemius muscle fibers. Data are expressed as mean ± SD (*n* = 6). **P < 0.01, *vs.* PLGA/CNT, ##*P* < 0.01, *vs.* PLGA/CNT-PDA, &*P* < 0.05, &&*P* < 0.01, *vs.* PLGA/CNT-PDA-NT_3_ (one-way analysis of variance followed by Tukey’s *post hoc* test). CMAP: Compound muscle action potential; CNT: carbon nanotube; NT_3_: neurotrophin-3; PDA: polydopamine; PLGA: polylactic-glycolic acid.

To further determine the impact of fibrous scaffolds on PNI repair, *in vivo* studies utilizing animal models were conducted, as illustrated in **Figure 6A**. The three-dimensional distribution of plantar pressure is depicted in **Figure 6B**. The results revealed that autograft was most effective, followed by PLGA/CNT-PDA-NT_3_. The SFI values across all groups exhibited similar trends, as mentioned above (**Figure 6C**). No significant variance was seen in SFI values between the PLGA/CNT and PLGA/CNT-PDA groups, whereas the SFI value of the PLGA/CNT-PDA-NT_3_ group was significantly higher (*P* < 0.05) than that of the other two groups. The lowest SFI value was observed in the autograft group (*P* < 0.01). Electrophysiological assessment of the gastrocnemius muscle (**Figure 6D** and **E**) indicated that the conduction velocity and peak amplitude of complex muscle action potential in the PLGA/CNT-PDA-NT_3_ group were superior to those in the PLGA/CNT (*P* < 0.01) and PLGA/CNT-PDA groups (*P* < 0.01).

Gastrocnemius muscle morphology is an indirect indicator of nerve regeneration. Prolonged denervation after sciatic nerve injury leads to muscle atrophy, characterized by a reduction in both the size and quantity of muscle fibers (Anzil and Wernig, 1989). As reinnervation progresses, atrophied muscle can gradually recover. Notably, gastrocnemius muscle atrophy was more frequently observed on the surgical side compared to the contralateral side in our model. Gastrocnemius atrophy was observed by the 12^th^ week. The wet weight of the muscles in the PLGA/CNT-PDA-NT_3_ group exceeded that of those in the PLGA/CNT-PDA group and the PLGA/CNT group (**Figure 6G**), suggesting less severe (*P* < 0.01) muscle atrophy in animals treated with PLGA/CNT-PDA-NT_3_ compared with the other groups. Gross photographs of the gastrocnemius muscle and cross-sectional Masson staining images are presented in **Figure 6F**. Blue staining signifies muscle fibrosis and collagen fiber deposition, indicating atrophy. Comparatively, the muscle fibers in the autograft and PLGA/CNT-PDA-NT_3_ groups exhibited fewer blue areas and thicker muscle fibers. Furthermore, Masson staining (**Figure 6H**) showed that the cross-sectional area of the gastrocnemius muscle fibers was significantly larger (*P* < 0.01) in the PLGA/CNT-PDA group than in the PLGA/CNT group and in the PLGA/CNT-PDA-NT_3_ group compared with the PLGA/CNT-PDA group (*P* < 0.01). In conclusion, the PLGA/CNT-PDA-NT_3_ scaffold significantly enhanced peripheral nerve regeneration and facilitated functional recovery *in vivo*.

### Polylactic-glycolic acid/carbon nanotube-polydopamine-neurotrophin-3–induced immune regulation *in vivo*

**Figure 7:**
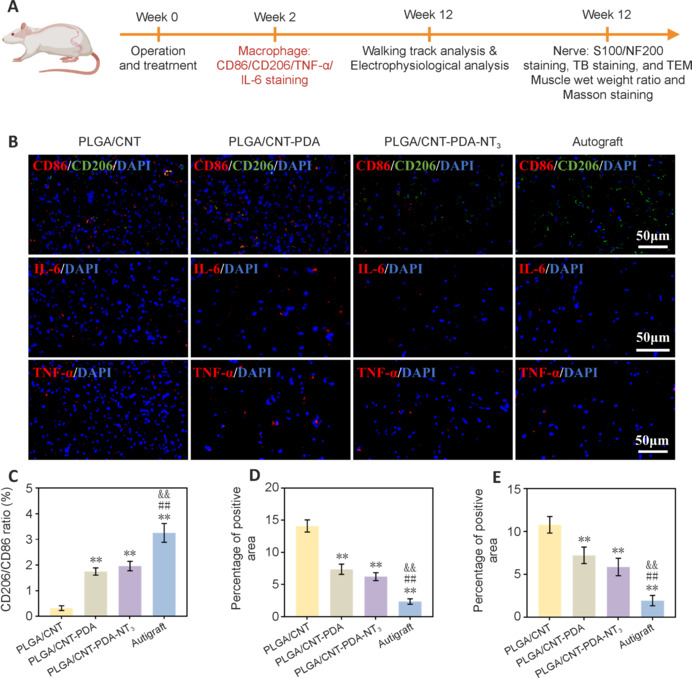
CD86, CD206, IL-6, and TNF-α immunofluorescence staining of the regenerated nerves. (A) Schematic diagram of wound healing in the rat sciatic nerve defect model. (B) CD86, CD206, IL-6, and TNF-α immunofluorescence staining (scale bars: 50 μm). (C) CD206/CD86 ratio, as calculated from the immunofluorescence staining. (D) IL-6 fluorescence-positive area. (E) TNF-α fluorescence-positive area. Data are expressed as mean ± SD (*n* = 6). **P < 0.01, *vs.* PLGA/CNT, ##*P* < 0.01, *vs.* PLGA/CNT-PDA, &&*P* < 0.01, *vs.* PLGA/CNT-PDA-NT_3_ (one-way analysis of variance followed by Tukey’s *post hoc* test). CNT: Carbon nanotube; IL: interleukin; NT_3_: neurotrophin-3; PDA: polydopamine; PLGA: polylactic-glycolic acid; TEM: transmission electron microscopy; TNF-α: tumor necrosis factor-α.

Macrophages are essential to the local immune response and inflammation after tissue injury. Inducing a macrophage phenotype shift from M1 to M2 could potentially expedite peripheral nerve regeneration, suppress inflammation, and foster nerve repair (Chen et al., 2015; Oshima et al., 2023; Yang et al., 2023). To explore this, immunofluorescence staining was conducted during the second week post-surgery, utilizing CD86 and CD206 as markers for M1 and M2 macrophages, respectively (**Figure 7A**). We observed a marked increase in the ratio of M2 to M1 macrophages in the PLGA/CNT-PDA-NT_3_ group compared with the PLGA/CNT and PLGA/CNT-PDA groups (*P* < 0.01; **Figure 7B** and **C**), suggesting that the PLGA/CNT-PDA-NT_3_ fibrous scaffold effectively facilitates the transformation of macrophages from an M1 phenotype to an M2 phenotype. **Figure 7B** shows that lower levels of TNF-α and IL-6 were detected in the PLGA/CNT-PDA-NT_3_ group compared with the other groups. Supporting this finding, the average IL-6–positive fluorescent area in the PLGA/CNT-PDA-NT_3_ (**Figure 7D**) was closest to that seen in the autograft group. The PLGA/CNT-PDA and PLGA/CNT groups exhibited significantly higher levels of IL-6 compared with the PLGA/CNT-PDA-NT_3_ group (*P* < 0.01). Similar results were observed for TNF-α immunofluorescence intensity (**Figure 7E**). These results demonstrate that the PLGA/CNT-PDA-NT_3_ fibrous scaffold induces macrophage phenotypic transformation from M1 to M2, reduces the expression of pro-inflammatory genes, suppresses excessive inflammation, and promotes nerve regeneration. As previously mentioned, the hydrophilicity and abundant functional groups introduced by PDA enhance cell adhesion and growth while scavenging ROS, while the electrical activity of CNT supports cell growth and development. The synergistic combination of these capabilities renders PLGA/CNT-PDA-NT_3_ a superior biological material in terms of ROS scavenging, immune regulation, and other composite functionalities.

### Histological analysis of regenerated nerve tissue

**Figure 8:**
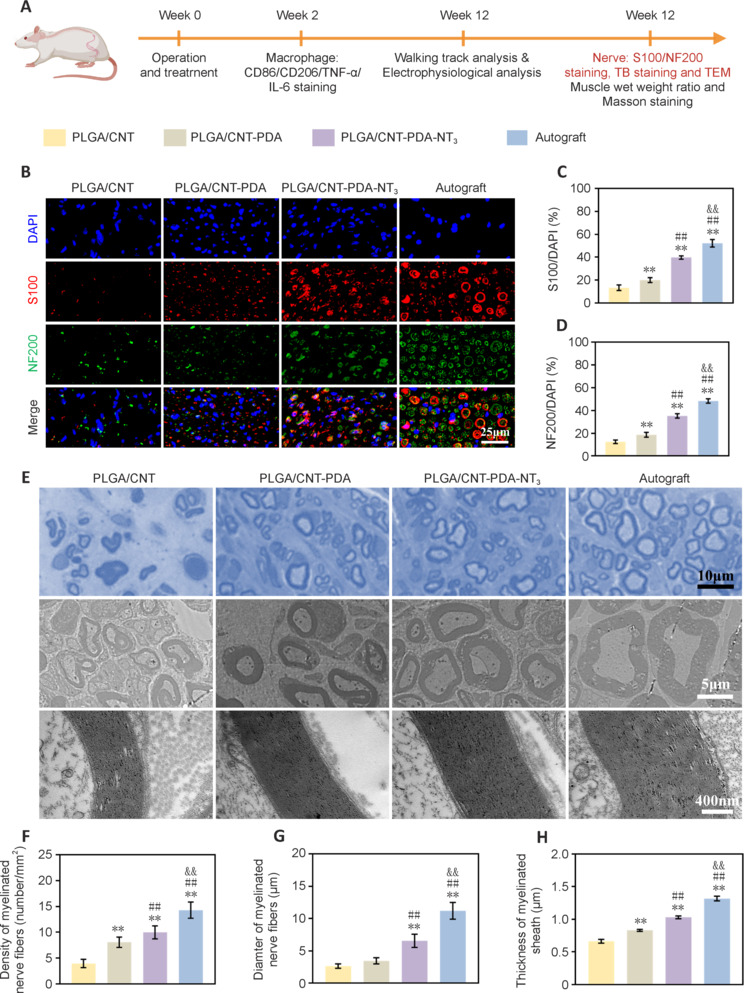
S100 and NF200 immunofluorescence staining, toluidine blue staining, and TEM imaging of the regenerated nerves. (A) Schematic diagram of wound healing in the rat sciatic nerve defect model. (B) S100/NF200 immunofluorescence staining (scale bar: 25 μm). (C) S100 fluorescence intensity. (D) NF200 fluorescence intensity. (E) Toluidine blue staining of regenerated nerve cross sections and TEM images of the myelin sheath (scale bars: 10 μm, 5 μm, 400 μm). (F) Average number of myelin sheaths per unit area. (G) Mean myelin diameter. (H) Mean myelin thickness. Data are expressed as mean ± SD (*n* = 10). **P < 0.01, *vs*. PLGA/CNT; ##*P* < 0.01, *vs.* PLGA/CNT-PDA; $$*P* < 0.01, *vs.* PLGA/CNT-PDA-NT3 group (one-way analysis of variance followed by Tukey’s *post hoc* test). CMAP: Compound muscle action potential; CNT: carbon nanotube; NF200: neurofilament-200; NT3: neurotrophin-3; PDA: polydopamine; PLGA: polylactic-glycolic acid; TEM: transmission electron microscopy.

Twelve weeks post-procedure, regenerated nerve sections were subjected to NF200 and S100 immunofluorescence staining (**Figure 8A**). The confocal cross-sectional images display NF200 and S100 protein as green and red, respectively. S100 expression was markedly higher (*P* < 0.01) in the PLGA/CNT-PDA-NT_3_ group *versus* the PLGA/CNT-PDA and PLGA/CNT groups, approaching the level observed in the autograft group (**Figure 8B** and **C**). A similar trend was observed for NF200 fluorescence. The average NF200 fluorescence intensity in the PLGA/CNT group was lower than that in the other groups (*P* < 0.01), while NF200 expression in the PLGA/CNT-PDA-NT_3_ group was close to that seen in the autograft group (**Figure 8B** and **D**).

To evaluate myelin regeneration after PNI, transverse semi-thin sections of regenerated nerve fibers were examined by toluidine blue staining (**Figure 8E**). The PLGA/CNT group exhibited fewer myelinated axons and thinner myelin sheaths compared with the other groups. Conversely, the PLGA/CNT-PDA-NT_3_ group and the autograft group displayed more myelinated axons and thick myelin sheaths, which exhibited a typical blue hollow-ring myelin structure. The degree of myelin regeneration in the PLGA/CNT-PDA-NT_3_ group closely resembled that seen in the autograft group, demonstrating superior outcomes compared with the other groups (**Figure 8E** and **F**). The average axon diameter in the PLGA/CNT group was smaller than that in the PLGA/CNT-PDA, PLGA/CNT-PDA-NT_3_, and autograft groups. The PLGA/CNT-PDA-NT_3_ group exhibited an average axon diameter closest to that seen in the autograft group (*P* < 0.01; **Figure 8G**). Additionally, as illustrated in **Figure 8H**, the myelin thickness in the PLGA/CNT-PDA-NT_3_ group significantly surpassed that seen in the PLGA/CNT-PDA and PLGA/CNT group (*P* < 0.01) and was the closest to seen in the autograft group. These results provide further evidence that fibrous scaffolds incorporating NT_3_ and PDA coating facilitate axon growth and myelination.

## Discussion

In this study, we developed a multifunctional drug-releasing nerve conduit and tested its performance in a rat model. The scaffold’s surface was modified by coating it with PDA, which was covalently linked to NT_3_, resulting in a novel bioactive scaffold. The PLGA/CNT-PDA-NT_3_ scaffold demonstrated excellent orientation, electrical conductivity, neurotrophic factor loading capacity, and sustained release properties. *In vitro* assessment showed excellent cell compatibility and growth-promoting effects, as well as an ability to scavenge ROS and regulate the immune response. *In vivo* experiments indicated that the scaffold effectively promoted nerve regeneration and functional recovery. Overall, the directed electroactive neural scaffold developed in this study exhibits good biocompatibility, antioxidant activity, and immunoregulatory properties, demonstrating strong potential for enhancing recovery from peripheral nerve injury.

We selected PLGA and CNTs to fabricate the scaffold using electrospinning technology and coated the scaffold with PDA and NT_3_ to enhance its functionality. PLGA, known for its distinctive physicochemical properties and exceptional biodegradability, has found extensive application in the formulation of long-acting drug delivery systems and has received approval from the US Food and Drug Administration (FDA) (Richner et al., 2014; Chen et al., 2015). CNTs are widely utilized in neural tissue engineering because of their well-organized fractal nanostructures and high electrical conductivity. Electrical signals positively regulate cellular behavior and facilitate intercellular communication among living cells, particularly nerve cells (Martino et al., 2012). Mechanical and electrical signals are both vital for initiating repair during nerve regeneration. CNTs have been extensively utilized in materials designed to promote tissue damage repair because of their excellent conductivity and electrical activity. Previous research has demonstrated that the high conductivity of CNTs can improve cell connections and boost the secretion of neurotrophic factors (Wu et al., 2022). PLGA/CNT fibrous scaffolds prepared using electrospinning technology have an oriented structure, which facilitates cell attachment, growth, and differentiation, thus effectively guiding the direction of neurite extension (Slejko et al., 2024). Additionally, because of the abundant functional groups in and polar chemical structure of PDA, the PDA coating has good hydrophilicity. In addition, the PDA coating can be covalently combined with active substances such as proteins through the Schiff base or Michael addition reaction to gain biological functions. Unlike electrostatic adsorption of proteins, which can result in sudden release, covalent binding is tighter, so the protein is gradually released as the coating degrades, achieving a prolonged release effect (Aleemardani et al., 2021; Muddineti and Omri, 2022).

Inflammation suppression and immune modulation have a great impact on nerve regeneration. PLGA/CNT-PDA-NT_3_ exhibited excellent anti-inflammatory and antioxidant properties toward RSC96, PC12, and RAW264.7 cells treated with H_2_O_2_, promoting macrophage polarization to an anti-inflammatory phenotype. The fibrous scaffolds modulated the local immune response and suppressed inflammation by reducing the secretion of inflammatory factors by macrophages through their antioxidant and ROS scavenging properties, thereby facilitating tissue repair. This may have been mediated by effective ROS scavenging by the PDA coating, thereby inducing a macrophage phenotype shift to the anti-inflammatory type and reducing the expression of inflammatory factors (Lagreca et al., 2020). In addition, CNT-mediated endogenous electrical signals may play a role in immune regulation (Huang et al., 2011; Tran et al., 2019).

Nerve regeneration is a complex process involving intricate cellular and biochemical cascades. The main mechanisms of repair include myelin regeneration, lateral branch budding, and axon growth. Axon regeneration failure can result from the absence of endogenous electrical signal conduction and structural disarray, while excessive inflammation can impede PNI repair. Therefore, key targets for accelerating regeneration after PNI include Schwann cell growth and migration, axon and neuron regeneration, and regulation of the inflammatory response. In this study, an oriented PLGA/CNT electroactive fibrous scaffold was developed using electrospinning technology, coated with PDA, and covalently to the neurotrophic factor NT_3_ to create a multifunctional nerve conduit. The remarkable capacity of PLGA/CNT-PDA-NT_3_ to promote PNI repair can be explained by a number of mechanisms. 1) The electrospun fibrous scaffold with an oriented structure mimics the natural extracellular matrix, providing topographic cues for cell survival and orderly growth. 2) Incorporation of PDA into the PLGA/CNT-PDA-NT_3_ scaffold enhances cell affinity, facilitating cell adhesion and growth. 3) The outstanding electrical properties of CNTs enable the fibrous scaffolds to connect biological activities at both ends of the damaged nerve, transmitting internal electrical signals and thereby promoting nerve regeneration. 4) The presence of catechol, amino, and other functional groups in PDA confers potent antioxidant and ROS scavenging capabilities, facilitating immune regulation, induction of macrophage M2 polarization, and inflammation inhibition in conjunction with transmission of endogenous electrical signals by the CNTs (Qiu et al., 2018). 5) Covalent coupling of the neurotrophic factor NT_3_ with the PDA coating provides sufficient nutrients for cells, accelerating cell proliferation and growth. Previous studies have found that neurotrophins bind to Trk receptors and mediate survival and differentiation through multiple pathways, including extracellular signal–regulated kinase, phosphoinositide 3-kinase, and phospholipase C-γ. Neurotrophins transmit survival signals via the p75NTR and NF-κB signaling pathways. In addition, neurotrophins regulate reorganization of the cytoskeleton and neurite growth by activating the downstream RhoA kinase (Bao et al., 2018). These mechanisms collectively target cells and the complex microenvironment surrounding the injury, thereby accelerating regeneration of damaged nerves.

This study has some limitations that should be noted. We did not perform any long-term evaluations or validate our findings in a large animal model, making it difficult to ensure the long-term effectiveness of the nerve conduits. Additionally, while electrospinning offers advantages such as controllable morphology, high surface area, and multifunctionality, it faces challenges related to large-scale production, consistency, and scalability. Precise control is required to generate uniform fiber morphology, and electrospun materials often fail quality assurance testing at high production capacities (Wu et al., 2023). Furthermore, several materials exhibit biological properties that are comparable to, or even superior to, CNTs. For example, graphene oxide (GO), a major derivative of graphene, has a large surface area, excellent thermal conductivity, high electrical conductivity, and a greater capacity for absorbing biomolecules (Gu et al., 2022). On the other hand, CNTs are a good biomaterial that is relatively affordable.

For future research, more representative experimental animals could be employed, such as pigs, to extend the duration of the experiments and observations, thereby obtaining more convincing evidence. The in-depth molecular mechanisms underlying nerve regeneration could be explored, such as the pathways through which PDA and endogenous electrical activity mediate immunomodulation and promote nerve regeneration. In addition, there are still many directions to be explored in terms of materials. Further investigations can be conducted in the future to identify materials that are multifunctional, highly safe, and cost-effective.

In conclusion, a multifunctional electrospun fibrous scaffold composed of PDA-coated PLGA/CNT composite and coupled with the neurotrophic factor NT_3_ was successfully developed in this study. The scaffold exhibits a well-defined oriented structure, electroactivity, efficient drug loading and sustained release, antioxidant properties, and potential for immune modulation. *In vitro* and *in vivo* evaluations demonstrated that the PLGA/CNT-PDA-NT_3_ scaffold effectively promotes nerve regeneration by stimulating Schwann cell proliferation, inducing secretion of nutrient factors, promoting neuronal differentiation, exerting anti-inflammatory responses, and inducing macrophage M2 polarization, among other underlying mechanisms. This study confirms that the PLGA/CNT-PDA-NT_3_ scaffold is an ideal neural conduit for tissue engineering and holds significant promise for applications in the treatment of peripheral nerve injury (PNI).

## Additional files:

***Additional Table 1:*** *Primers used in this study for quantitative reverse transcription-polymerase chain reaction.*

***Additional Figure 1:*** *The tensile strength of PLGA/CNT, PLGA/CNT-PDA and PLGA/CNT-PDA-NT*_*3*_ *scaffolds.*

Additional Figure 1 The tensile strength of PLGA/CNT, PLGA/CNT-PDA and PLGA/CNT-PDA-NT3 scaffolds.
Data are expressed as mean ± SD (*n =* 3). CNT: Carbon nanotube; NT3: neurotrophin-3; PDA: polydopamine; PLGA: polylactic-glycolic acid.

***Additional Figure 2:*** *Degradation rate of PLGA/CNT, PLGA/CNT-PDA and PLGA/CNT-PDA-NT*_*3*_ *scaffolds in vitro.*

Additional Figure 2 Degradation rate of PLGA/CNT, PLGA/CNT-PDA and PLGA/CNT-PDA-NT3 scaffolds *in vitro*.
Data are expressed as mean ± SD (*n* = 3). *P < 0.05, *vs.* PLGA/CNT. CNT: Carbon nanotube; NT3: neurotrophin-3; PDA: polydopamine; PLGA: polylactic-glycolic acid.

## Data Availability

*All relevant data are within the paper and its Additional files*.
