# Supplementary figures and images for: Polydopamine-coupled NT3-derived oriented conductive scaffolds with immunomodulatory properties accelerate peripheral nerve regeneration
Source: Neural Regen Res. 2025 Sep 3;21(6):2658–68. doi: 10.4103/NRR.NRR-D-24-01544 (PMC13211852; doi:10.4103/NRR.NRR-D-24-01544)

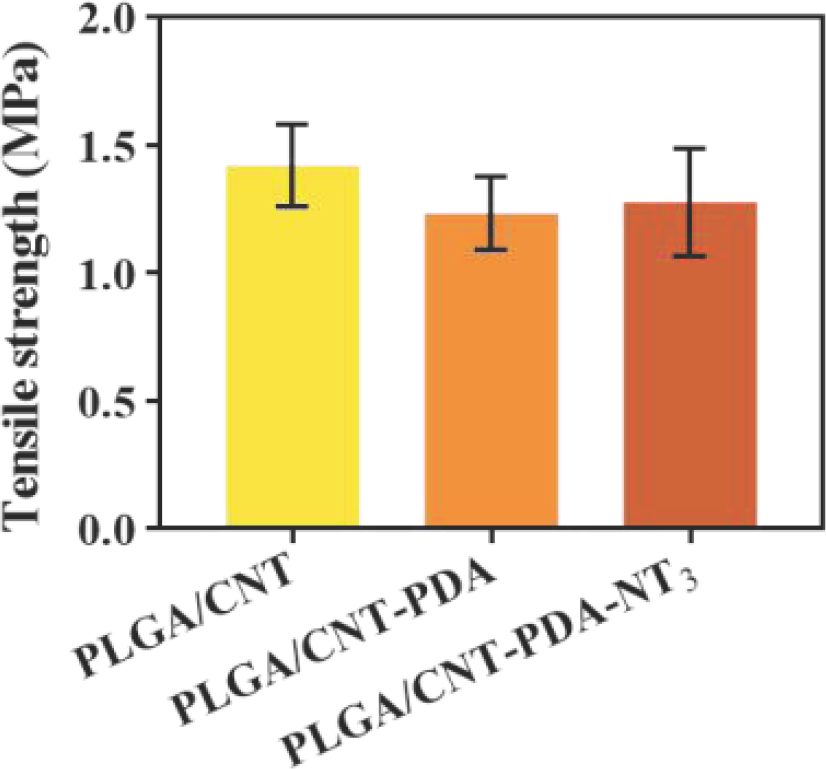

Supplement: Supplementary file 1 [file NRR-21-2658_Suppl1.tif]

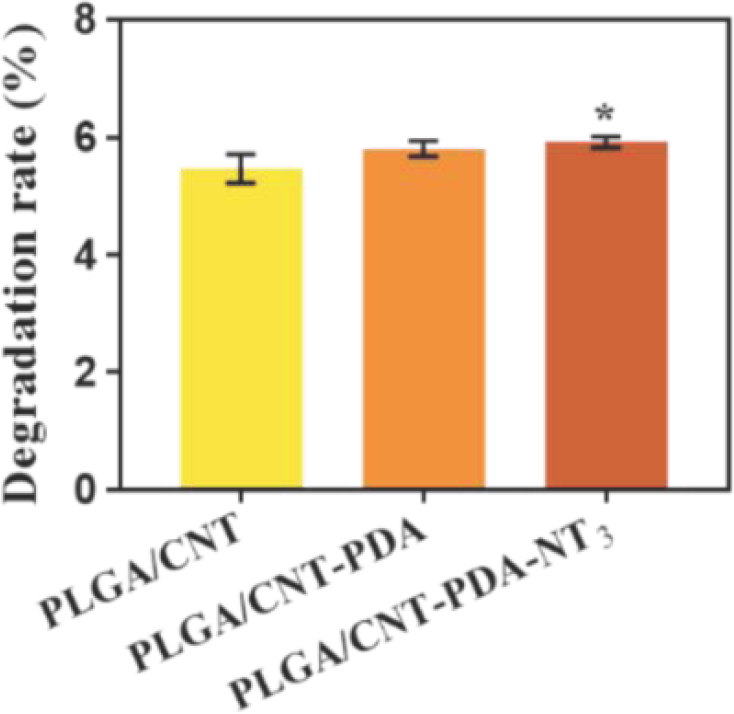

Supplement: Supplementary file 2 [file NRR-21-2658_Suppl2.tif]
